# Diabetes and Insulin Injection Modalities: Effects on Hepatic and Hippocampal Expression of 11β-Hydroxysteroid Dehydrogenase Type 1 in Juvenile Diabetic Male Rats

**DOI:** 10.3389/fendo.2017.00081

**Published:** 2017-04-18

**Authors:** Véronica Rougeon, Marie-Pierre Moisan, Nicole Barthe, Marie-Christine Beauvieux, Jean-Christophe Helbling, Véronique Pallet, Nathalie Marissal-Arvy, Pascal Barat

**Affiliations:** ^1^Unité d’endocrinologie et de diabétologie pédiatrique, CHU de Bordeaux, Hôpital des Enfants, Bordeaux, France; ^2^INRA, Laboratoire de Nutrition et Neurobiologie Intégrée, UMR 1286, Bordeaux, France; ^3^University of Bordeaux, Nutrition et neurobiologie intégrée, UMR 1286, Bordeaux, France; ^4^Laboratoire mixte de Biophysique – INSERM U1026 BioTis, Bordeaux, France; ^5^Laboratoire de Biochimie de l’Hôpital Haut-Lévêque, Pessac, France; ^6^Bordeaux INP, Talence, France

**Keywords:** type 1 diabetes, glucocorticoids, 11β-hydroxysteroid dehydrogenase type 1, insulin, liver, hippocampus

## Abstract

**Background:**

Dysregulation of the hypothalamic–pituitary–adrenal (HPA) axis is often encountered in diabetes, leading to several clinical complications. Our recent results showing an elevated tetrahydrocortisol/tetrahydrocorticosterone ratio in morning urine of diabetic children compared to that of controls suggest an increased nocturnal activity of 11β-hydroxysteroid dehydrogenase type 1 (11β-HSD1) in the former.

**Question:**

We hypothesized that these observations could be explained by a reduced inhibition of hepatic 11β-HSD1 activity by exogenous insulin owing to its subcutaneous (SC) administration and absence of first hepatic passage. Additionally, we hypothesized that hippocampal 11β-HSD1 activity might also be impaired by diabetes.

**Methods:**

We therefore measured HPA axis activity and 11β-HSD1 expression and activity in liver and hippocampus in streptozotocin-induced diabetic juvenile rats treated with SC or intraperitoneal (IP) insulin.

**Results:**

Plasma corticosterone levels were elevated in untreated diabetic rats during the resting phase and restored by both types of insulin treatment. The mRNA expression and activity of 11β-HSD1 were increased in the untreated diabetic group in liver. Although diabetes was controlled equally whatever the route of insulin administration, liver 11β-HSD1 gene expression and activity was decreased only in the IP group, suggesting that a first hepatic pass is needed for 11β-HSD1 hepatic inhibition. In hippocampus, 11β-HSD1 activity was elevated in the untreated diabetic group but restored by both types of insulin treatment. Thus, these data extend our findings in diabetic children by showing impairment of hippocampal 11β-HSD1 in diabetes and by demonstrating that IP is preferable to SC insulin administration to restore 11β-HSD1 activity in liver.

## Introduction

The incidence of type 1 diabetes mellitus (T1D) in children is increasing worldwide, particularly in children under 5 years of age (1). Patients and animals with poorly controlled diabetes show dysregulation of their hypothalamic–pituitary–adrenal (HPA) axis (2), and this dysfunction can be involved in complications of diabetes, such as cognitive impairment (3). To further understand the impairment of the HPA axis that occurs in diabetes, we recently studied the nocturnal tetrahydrocortisol/tetrahydrocorticosterone (THFs/THE) ratio in children with T1D since it reflects 11β-hydroxysteroid dehydrogenase type 1 (11β-HSD1) activity in most cases (4). The 11β-HSD1 enzyme plays a crucial role in the bioavailability of glucocorticoids by regenerating inactive cortisone into active cortisol in humans and inactive dehydrocorticosterone into corticosterone in rodents (5). Our recent study suggested an increased nocturnal activity of 11β-HSD1 in diabetic children compared to controls, since THFs/THE ratio was elevated in the morning urine of diabetic children (4). One possible explanation for this is a reduced inhibition of hepatic 11β-HSD1 activity by exogenous insulin (6) owing to its subcutaneous (SC) route and thus the absence of a first hepatic pass. To test this hypothesis, we measured the HPA axis activity and 11β-HSD1 liver expression and activity in streptozotocin-induced diabetic juvenile rats treated with either SC or intraperitoneal (IP) insulin. As HPA axis has been suggested to be involved in cognitive and emotional disorders in T1D patients (7, 8), and hippocampal 11β-HSD1 to be implicated in neurological disorders (9, 10), we also explored the effects of our treatments on the expression and activity of 11β-HSD1 in hippocampus.

## Materials and Methods

### Experimental Design

Male Wistar rats were purchased from Janvier (Saint Berthevin, France). Weaning rats (3-week-old, 50–55 g, *n* = 48) were housed four per cage in a room with a constant airflow system and controlled temperature (21–23°C) and were weighed throughout the protocol. They were acclimatized for 7 days to a reverse 12-h light cycle (lights off at 10:00 a.m.) in order to administer insulin during the active feeding phase and to avoid hypoglycemia. Rats (*n* = 36) were treated with streptozotocin on day 0 (IP 65 mg/kg, citrate buffer 0.1 M pH 4.5). The 12 remaining rats formed the control group (0.1 ml IP injection of vehicle). Blood glucose was measured 3 days after streptozotocin injection (Glucometer Freestyle, Abbott Diabetes Care Ltd., Alameda, CA, USA), and rats that showed hyperglycemia >200 mg/dl were considered diabetic. These rats were then split into 3 groups of 12 individuals: a group of untreated diabetics (diabetics NT), a diabetic group treated with SC insulin (Lantus^®^, insulin glargine from Sanofi, Paris, France), and a diabetic group treated with IP insulin. Doses of insulin were adapted to the glycemic response of each animal. At the end of the experiment, in the morning, 1 week after glycemia control had been obtained, animals were killed by decapitation. Liver and hippocampus were collected from each rat and immediately frozen at −80°C. In order to measure plasma corticosterone, one blood sample was collected once in the morning at 10:00 a.m. (end of the resting period) and once in the evening at 10:00 p.m. (end of the active period) 2 days before sacrifice. Blood was collected in EDTA-coated tubes and centrifuged at 4,000 × *g* for 10 min at 4°C in order to obtain plasma, which was stored at −80°C.

### Plasma Fructosamine

Plasma fructosamine concentration to assess short-term glycemic control was measured by using a glycated serum protein test as described in Ref. (11). Fructosamine is a marker for glycemic control in short term (2–3 weeks). Concentration was measured using an enzymatic method RANDOX, with proteinase K digesting glycated serum proteins into glycated peptide fragments (GPF). This method also uses a specific fructosaminase (fructosylamino-oxydase) to catalyze oxidative degradation of GPF into protein fragments or amino-acids, glucosone, and hydrogen peroxide. H_2_O_2_ is measured using a Trinder colorimetric reaction, and absorbance is proportional to sample’s glycated protein concentration.

### Plasma Corticosterone

Plasma corticosterone was measured with an in-house radioimmunoassay as described in Ref. (12), using a highly specific antibody provided by H. Vaudry (University of Rouen, France). Briefly, after steroid extraction of plasma samples with absolute ethanol, total corticosterone was measured by competition between cold corticosterone and ^3^H-corticosterone for the specific anti-corticosterone antibody.

### 11β-HSD1 Gene Expression (RT-PCR)

Total RNA was extracted from liver and hippocampus and reverse-transcribed as detailed before (13). RNA was extracted with a Trizol^®^ extraction kit (Invitrogen, Saint Aubin, France) according to the manufacturer’s recommendations. Samples in a final volume of 50 µl were treated with DNAse (Turbo DNA free kit, Ambion, Invitrogen) for 20 min at 37°C. RNAs were quantified by spectrophotometry using a Nanodrop 1000 Thermo Scientific, and their quality was assessed on Agilent RNA 6000 nano chips with an Agilent 2100 Bioanalyzer. Reverse transcription took place on aliquots of total RNA of 2 µg using a Superscript III Invitrogen kit containing oligohexameres, dNTP, and sterile water. RNA was denatured by heating at 65°C for 5 min. RT reactions were conducted in a buffer at 50°C for 55 min and terminated by heating at 85°C for 5 min followed by cooling at 4°C. mRNA levels were measured in real-time PCR using SYBR^®^ green 1 as the fluorescent marker. Sequences of primers for the 18S housekeeping gene were forward 5′ACCGCAGCTAGGAATAATGGA3′ and reverse 5′GCCTCAGTTCCGAAAACCAA3′, and for the 11β-HSD1 gene, forward 5′TGGAAGACATGGCTTTTGCA3′ and reverse 5′TCCAGTCCACCCAAGAGCTT3′. The relative mRNA expres-sion level of the target gene was expressed as 2^−ΔΔCT^ in comparison with the mean ΔCT of the control group.

### 11β-HSD1 Activity

11β-hydroxysteroid dehydrogenase type 1 activity was measured in rat liver and hippocampus homogenates obtained and treated as described by others (14). 11β-HSD1 activity was measured in rat liver and hippocampus homogenates, obtained by grinding the samples with a tissue-lyser in a buffer containing protease and phosphatase inhibitors [glycerol (1.37 M), NaCl (300 mM), EDTA (1 mM), Tris (50 mM), PIC 1X (protease inhibitor cocktail), NaOV (2 mM), and NaF (1 mM), pH 7.7]. Total quantity of proteins of the homogenate was then measured by colorimetry with a BCA protein assay (Bio-Rad protein assay kit) and a 560 nm microplaque spectrophotometer (Wallac 1420 VICTOR3). Aliquots of each homogenate were prepared with a protein concentration of 0.1 mg/ml. Aliquots were then incubated for 30 min at 37°C with 100 nM of tritium-marked corticosterone (Perkin Elmer) and an excess of the 11β-HSD-specific cofactor NADP+ (Sigma, 2 mM for liver, 400 µM for hippocampus). Samples were put on ice, and steroids were extracted with ethyl acetate. Samples were then dried in a Speedvac at 50°C for 30 min and dissolved in 60 µl ethanol. Steroids were separated by thin-layer chromatography (TLC Silica Gel 60 F254, VWR) with chloroform and ethanol as the mobile phase (92% chloroform, 8% ethanol). Two steroids were separated: ^3^H-corticosterone (substrate, B) and ^3^H-dehydrocorticosterone (product, A). Fractional conversion of steroids was calculated after scanning analysis using a β-IMAGER-2000 Phosphor Image Scanner in the same time as an increasing scale of tritium corticosterone concentrations (0.1–100 nCi). Steroids were identified comparing their migration and quantified using βVISION+ software program. 11β-HSD1 reductase activity was expressed as the ratio of dehydrocorticosterone/(corticosterone + dehydrocorticosterone).

### Data Analysis

Data were analyzed with Statistica software. Results were expressed as means ± SEMs. Body weight was analyzed by a two-way ANOVA with treatment as the between factor and time as the repeated factor. Other data were analyzed by a one-way ANOVA (between factor: treatment). Subsequently, when ANOVA was significant (*p* < 0.05), Fisher *post hoc* analyses were conducted.

## Results

### Diabetes Induction and Insulin Control

**Figure 1:**
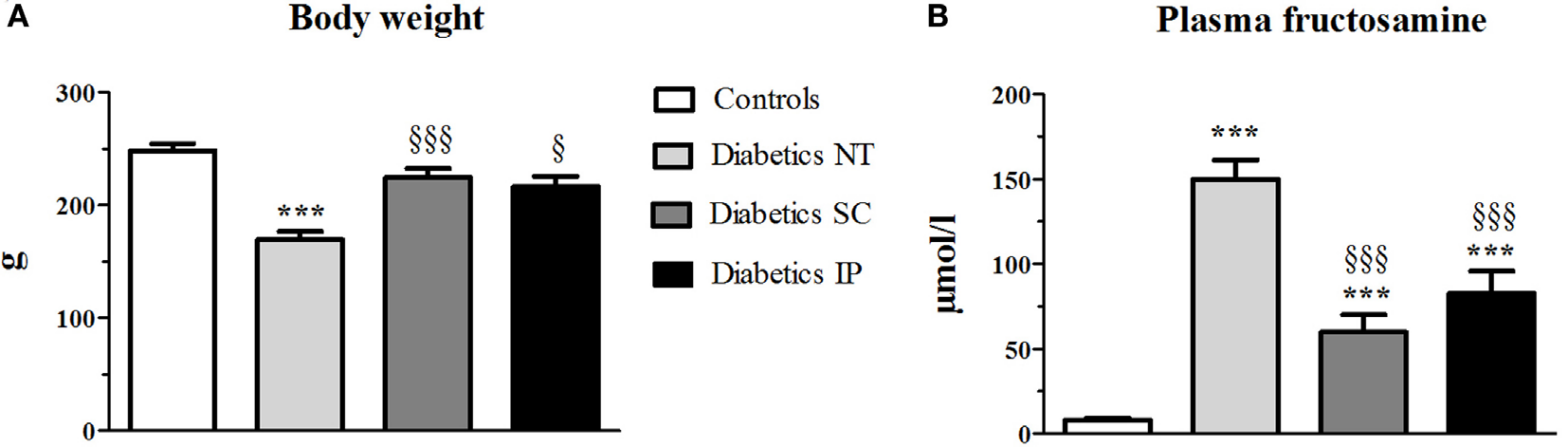
**Body weight at the end of the experiment (A) and plasma fructosamine at sacrifice (B)**. Diabetics NT, untreated diabetic rats; Diabetics SC, diabetic rats treated with subcutaneous insulin; Diabetics IP, diabetic rats treated with intraperitoneal insulin. Mean ± SEM, ***p < 0.001 compared to the control group, ^§^*p* < 0.05, ^§§§^*p* < 0.001 compared to the NT group.

The dose of insulin received was not different between diabetic SC and IP rat groups throughout the experiment (3.29 ± 0.61 vs. 3.86 ± 0.90 UI/day, respectively, per group at the end of the experiment, NS). At the end of the experiment, the diabetic NT rats showed a lower weight than controls (*p* < 0.05, globally). SC and IP insulin administration normalized the weight of diabetic NT rats (Figure 1A).

Fructosamine increased in the diabetic NT, SC, and IP groups compared to the control group (*p* < 0.001, Figure 1B). Both insulin treatments led to a significant decrease in fructosamine compared to the diabetic NT group (*p* < 0.001).

### Plasma Corticosterone

**Figure 2:**
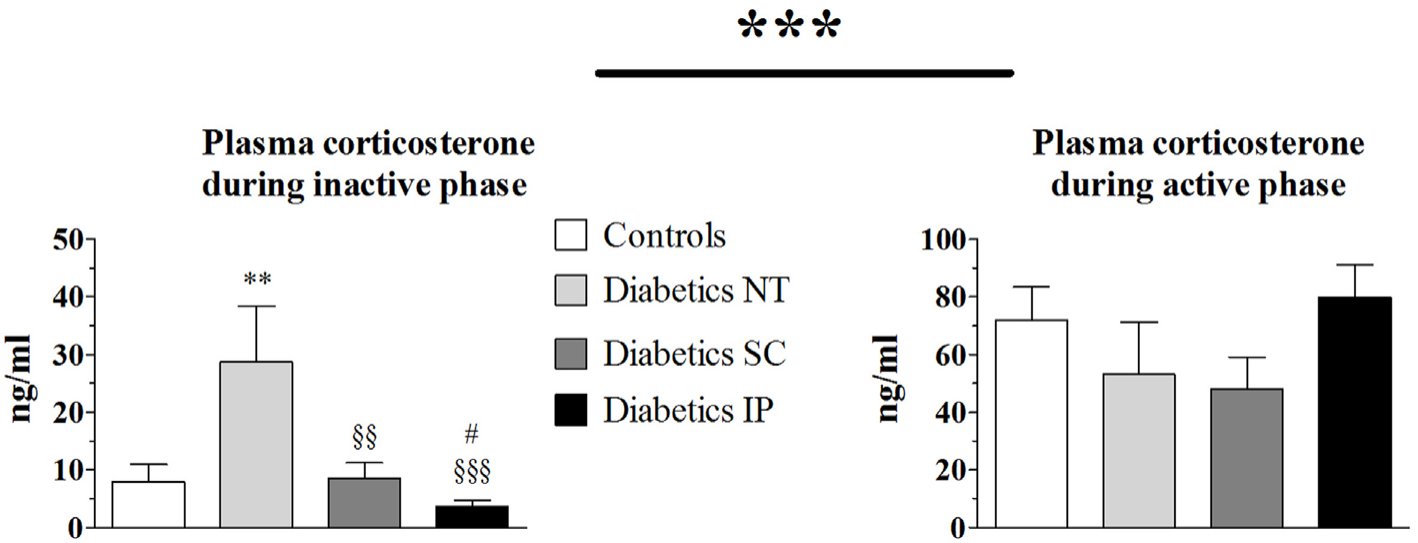
**Plasma corticosterone**. Diabetics NT, untreated diabetic rats; Diabetics SC, diabetic rats treated with subcutaneous insulin; Diabetics IP, diabetic rats treated with intraperitoneal insulin. Mean ± SEM, **p < 0.01 compared to the control group, ^§§^*p* < 0.01, ^§§§^*p* < 0.001 compared to the NT group, ^#^*p* < 0.05 compared to the subcutaneous group.

Total corticosterone increased during the active phase compared to the resting (inactive) phase (*p* < 0.001) in all groups (Figure 2). Corticosterone was elevated in the diabetic NT group during the resting phase compared to the control group (*p* < 0.05). This increase was normalized in the diabetic SC (*p* < 0.01) and IP (*p* < 0.001) groups. Corticosterone was lower in the diabetic IP group than in the SC group (*p* < 0.05). No significant difference in corticosterone levels between groups were found by ANOVA during the active phase.

### Liver 11β-HSD1 Gene Expression and Activity

**Figure 3:**
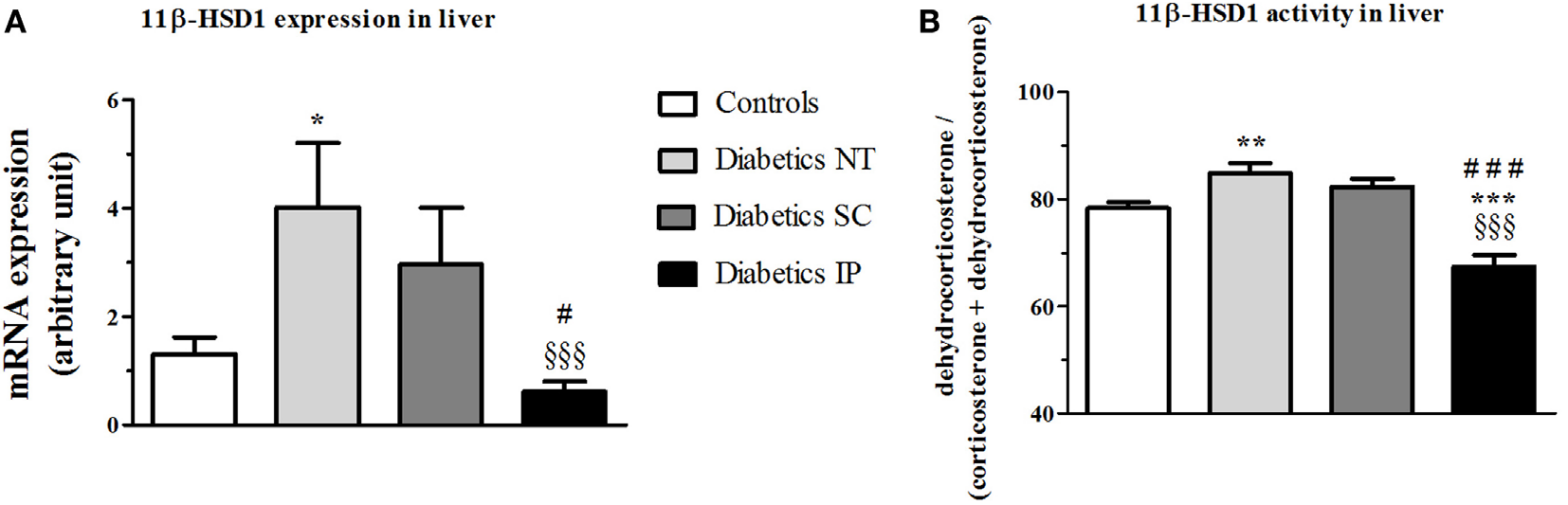
**11β-HSD1 expression and activity (percent conversion) in liver**. 11β-HSD1, 11β-hydroxysteroid dehydrogenase type 1; Diabetics NT, untreated diabetic rats; Diabetics SC, diabetic rats treated with subcutaneous insulin; Diabetics IP, diabetic rats treated with intraperitoneal insulin. Mean ± SEM, *p < 0.05, **p < 0.01, ***p < 0.001 compared to the control group, ^§§§^*p* < 0.001 compared to the NT group, ^#^*p* < 0.05, ^###^*p* < 0.001 compared to the subcutaneous group. **(A)** 11β-HSD1 expression in liver. **(B)** 11β-HSD1 activity in liver.

ANOVA revealed a strong main effect of treatment (*p* < 0.001) on 11β-HSD1 gene expression in liver (Figure 3). The diabetic NT group showed a significant increase in 11β-HSD1 liver mRNA expression (*p* < 0.05), which was significantly lowered in the diabetic IP group compared to the diabetic NT and SC groups (*p* < 0.001 and *p* < 0.05, respectively). The diabetic SC group presented a higher variability with 11β-HSD1 liver mRNA expression intermediate between controls and NT diabetic rats and not significantly different from any of these groups (Figure 3A). The same pattern was found for 11β-HSD1 activity in liver. Diabetic NT rats showed increased liver 11β-HSD1 activity vs. the control group (*p* < 0.01). Diabetic IP group had lower 11β-HSD1 activity compared to the other groups (*p* < 0.001), while diabetic SC rats were not different from controls and diabetic NT (Figure 3B).

### Hippocampus 11β-HSD1 Gene Expression and Activity

**Figure 4:**
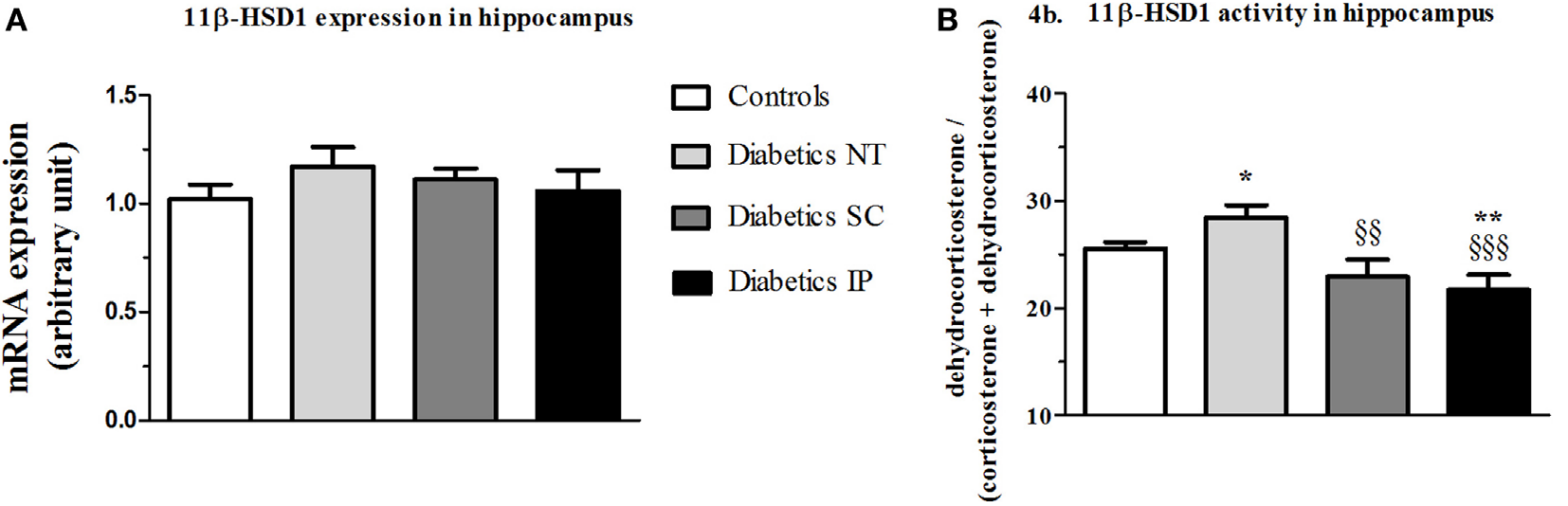
**11β-HSD1 expression and activity (percent conversion) in hippocampus**. 11β-HSD1, 11β-hydroxysteroid dehydrogenase type 1; Diabetics NT, untreated diabetic rats; Diabetics SC, diabetic rats treated with subcutaneous insulin; Diabetics IP, diabetic rats treated with intraperitoneal insulin. Mean ± SEM, *p < 0.05, **p < 0.01, when compared to the control group, ^§§^*p* < 0.01, ^§§§^*p* < 0.001 when compared to the NT group. **(A)** 11β-HSD1 expression in hippocampus. **(B)** 11β-HSD1 activity in hippocampus.

There was no statistical difference in hippocampal 11β-HSD1 mRNA expression between the groups (Figure 4A). However, 11β-HSD1 activity measured in hippocampus showed a significant main treatment effect (*p* < 0.01). Diabetic NT rats presented an increased 11β-HSD1 activity (*p* < 0.05) compared to controls, which was normalized by insulin treatments more significantly in the IP (*p* < 0.001) than in the SC groups (*p* < 0.01) (Figure 4B).

## Discussion

Following on from our previous study in children with diabetes, we examined in the present study possible impairments of corticosterone levels and of liver 11β-HSD1 in diabetic juvenile rats. We also studied the effect of different route of insulin administration on 11β-HSD1 expression and activity with the hypothesis that SC insulin administration is not efficient to inhibit 11β-HSD1 in liver because of the absence of first hepatic passage. Finally, we explored 11β-HSD1 in hippocampus because hippocampal 11β-HSD1 is known to be involved in cognitive impairment during aging (9) and thus might play also a role in the cognitive impairments associated with T1D.

As expected, untreated diabetic rats have a lower weight and higher glycemia than controls, associated with increased corticosterone levels during the resting phase and higher 11β-HSD1 mRNA and activity in liver measured at the end of the resting phase. Lack of insulin is known to lead to a hypercatabolic state illustrated by the failure to thrive. This stress probably activates the HPA axis centrally and increases plasma corticosterone levels. Increased liver 11β-HSD1 activity probably also contributes to the elevated corticosterone levels in diabetic rats.

Interestingly, while no significant differences were observed during the active phase, the resting phase appeared to be more sensitive for differentiating HPA axis activity between the groups. Furthermore, increased 11β-HSD1 activity during the resting phase is in line with our findings in diabetic children in whom 11β-HSD1 activity was found increased in urine collected during the night (4). Since others did not find any difference over 24 h of urine collection (15), these findings in children and in the present animal study underline the need to take into account the phase of the nycthemeral cycle in which HPA axis activity is studied. Functionally, increased corticosterone levels at time of secretion nadir results in a globally flattened circadian secretion of the hormone that is known to have deleterious consequences on the body as well as on the brain (16). Of note, hippocampal 11β-HSD1 activity was also found elevated in diabetic untreated animals. Since 11β-HSD1 increases local corticosterone levels, it may further contribute to cognitive impairments in T1D in addition to the lack of insulin and to dysregulated systemic corticosterone levels.

Although we obtained the same level of glycemic control of diabetes, assessed by glucose and fructosamine levels, between the SC vs. IP insulin-treated groups, 11β-HSD1 gene expression and activity were significantly decreased only in the IP insulin-treated group in liver. This result supports our hypothesis that a first hepatic pass may be needed for efficient 11β-HSD1 hepatic inhibition, which would not be achieved efficiently by SC insulin treatment. Further investigations are required to prove the hypothesis. Furthermore, the effects of insulin could be indirect, for instance, by being mediated by an increase in growth hormone (GH) activity, which was described to inhibit 11β-HSD1 activity (17), whereas insulin increases levels of GH receptors (18). By reducing the hypercatabolic state and then limiting stress-induced HPA axis central activation, SC insulin treatment normalized plasma corticosterone levels in the resting state. Interestingly, IP insulin treatment decreased plasma corticosterone levels even more than SC. This effect could be linked in part to the inhibition of hepatic 11β-HSD1 activity that is clearly more efficient in IP vs. SC insulin administration. This finding could further support the use of IP insulin delivery that has shown numerous benefits in other studies (lower peripheral insulinemia, better reproducibility of insulin profiles, and improved glucagon response to hypoglycemia) owing to a pharmacokinetic that is closer to the physiological state than the SC route (19).

In conclusion, the present findings extend our data reported in diabetic children in that diabetes leads to an increase of 11β-HSD1 activity not only in liver but also in hippocampus. Our data suggest a new potential benefit of the IP route of insulin treatment. By better limiting the regeneration of active glucocorticoids in the liver of diabetic patients, it could also have a positive impact on complications that occur as a result of dysregulation of the corticotropic axis.

## Ethics Statement

All animal experiments were conducted according to the INRA Quality Reference System and to relevant French (Directive 87/148, Ministère de l’Agriculture et de la Pêche) and international (Directive 86/609, November 24th 1986, European Community) legislation. They followed procedures approved by Région Aquitaine Veterinary Services (Direction Départementale de la Protection des Animaux, approval ID: A33-063-920). Our local ethics committee specifically approved this study (Comité d’Ethique de Bordeaux, agreement #5012063-A).

## Author Contributions

NM-A: manipulations, organization, and redaction. VR, NB, M-CB, and J-CH: manipulations. M-PM and PB: organization and redaction. VP: redaction.

## Conflict of Interest Statement

The authors declare that there is no conflict of interest that could be perceived as prejudicing the impartiality of the research reported.
